# Cronkhite-Canada syndrome after corticosteroid and mesalazine treatment: A case report and 3-year follow-up

**DOI:** 10.1097/MD.0000000000047136

**Published:** 2026-01-16

**Authors:** Rui-Rui Yang, Wei Jiang, Yu-Feng Li

**Affiliations:** a Department of Gastroenterology, The First Clinical College (Affiliated Hospital of Liaoning University of Traditional Chinese Medicine), Liaoning University of Traditional Chinese Medicine, Shenyang, Liaoning Province, China; b Department of Gastroenterology, Affiliated Hospital of Liaoning University of Traditional Chinese Medicine, Shenyang, Liaoning Province, China.

**Keywords:** case report, Cronkite-Canada syndrome, gastrointestinal polyposis, prognosis

## Abstract

**Rationale:**

Cronkhite-Canada syndrome (CCS) is a rare, nonhereditary gastrointestinal polyposis syndrome with known malignant potential. This case report aims to present the clinical course, management, and long-term (3-year) follow-up of a CCS patient, highlighting the discrepancy between symptomatic improvement and endoscopic progression, and to discuss associated carcinogenic risk.

**Patient concerns:**

A 50-year-old man presented with diffuse abdominal pain of unclear origin, bloody stools, hair loss, melanosis on the hands, and nail dystrophy.

**Diagnoses:**

Laboratory tests revealed hypoalbuminemia, hypocalcemia, hypokalemia, and positive fecal occult blood. Enteroscopy and subsequent pathological examination confirmed the presence of characteristic intestinal polyps, leading to a diagnosis of CCS.

**Interventions:**

The patient was treated with a continuous regimen of corticosteroids combined with mesalazine. After 1 year of medical therapy, he underwent endoscopic mucosal resection.

**Outcomes:**

The patient’s clinical symptoms improved significantly following treatment. However, endoscopic and pathological findings showed a tendency to deteriorate over time. At the 3-year follow-up, this disparity between clinical well-being and pathological progression persisted.

**Lessons:**

CCS carries a risk of malignant transformation. Even with clinical improvement on long-term corticosteroid and mesalazine therapy, endoscopic progression may occur. Therefore, we recommend intensive endoscopic surveillance every 3 to 6 months following initial diagnosis, maintained for at least the 1st year, as a strategy to mitigate cancer risk.

## 1. Introduction

Cronkhite-Canada syndrome (CCS) is characterized by ectodermal abnormalities and diffuse polypoid lesions of the gastrointestinal tract with protein loss. It is distinguished by widespread gastrointestinal polyposis, protein-losing enteropathy, diarrhea, and 3 dermatological conditions: alopecia, urticaria, and hyperpigmentation.^[1,2]^ There is no consensus on treatment; the most recommended treatment for CCS is long-term corticosteroids, but mesalazine has been shown to have good efficacy for intestinal lesions as well. Due to the tendency of CCS to become malignant, regular endoscopy is an effective measure to prevent cancer.

## 2. Case Reports

### 2.1. Case presentation

**Figure 1.**
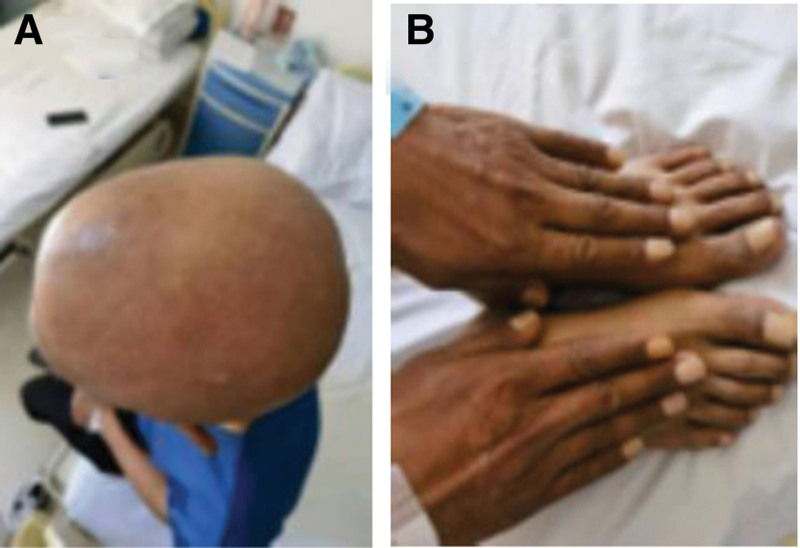
Hair loss (A), hyperpigmentation of the skin in the upper limbs (B).

In August 2022, a 50-year-old male patient was admitted to the hospital with abdominal pain, hair loss, nail atrophy, and skin hyperpigmentation of the mouth and hands as the main symptoms (Fig. 1A and B). The patient’s history revealed watery stools 4 to 5 times a day for the past 1 month, accompanied by the presence of bloody stools and mild limb twitching. The patient did not have any underlying medical conditions such as diabetes mellitus or hypertension, and there was no history of surgery, medications, or allergies. The personal history included a history of alcohol consumption for more than 30 years, which had been abstained from for 2 years, and a history of smoking for more than 30 years, with about 20 cigarettes per day. In family history, Father had bone cancer.

### 2.2. Physical examination

T: 36.2°C, P: 80 beats/min, R: 18 breaths/min, BP: 110/75 mm Hg; The patient had no yellow staining of the skin and mucous membranes, no hemorrhagic spots, no rash, and no enlargement of the superficial lymph nodes, but the skin of both hands was dark brown, and the nails were pale, thin, and cracked at the nail bed. The head is normal in size and without deformity, but there is alopecia and diffuse hyperpigmentation.

### 2.3. Laboratory examinations

Laboratory results showed that the patient did not meet the criteria for anemia, although bloody stools were present. Total protein and albumin were decreased, suggesting that the patient was recently malnourished, which could also be the cause of protein-losing enteropathy. The patient had recently developed renal insufficiency with elevated urinary transferrin, urinary β2-microglobulin, and urinary α1-microglobulin, suggesting early glomerular injury (Table 1).

**Table 1:** Laboratory findings.

Parameters	Findings	Normal range	Remarks
Hemoglobin (g/L)	142	120–160	Normal
Protein (g/L)	51.52	66–87	Low
Albumin (g/L)	29.19	34–48	Low
Albumin to globulin ratio	1.3	1.5–2.5	Low
Immunoglobulin A (g/L)	1.72	2.01–2.69	Low
Immunoglobulin G (g/L)	8.90	11.52–14.22	Low
Immunoglobulin M (g/L)	0.39	0.84–1.32	Low
Potassium (mmol/L)	3.4	3.5–5.5	Low
Chloride (mmol/L)	110	100–108	High
Calcium (mmol/L)	1.95	2.15–2.57	Low
Phosphorus (mmol/L)	0.82	0.96–1.62	Low
Fecal occult blood test	Positive	Negative	Positive

### 2.4. Imaging examinations

**Figure 2.**
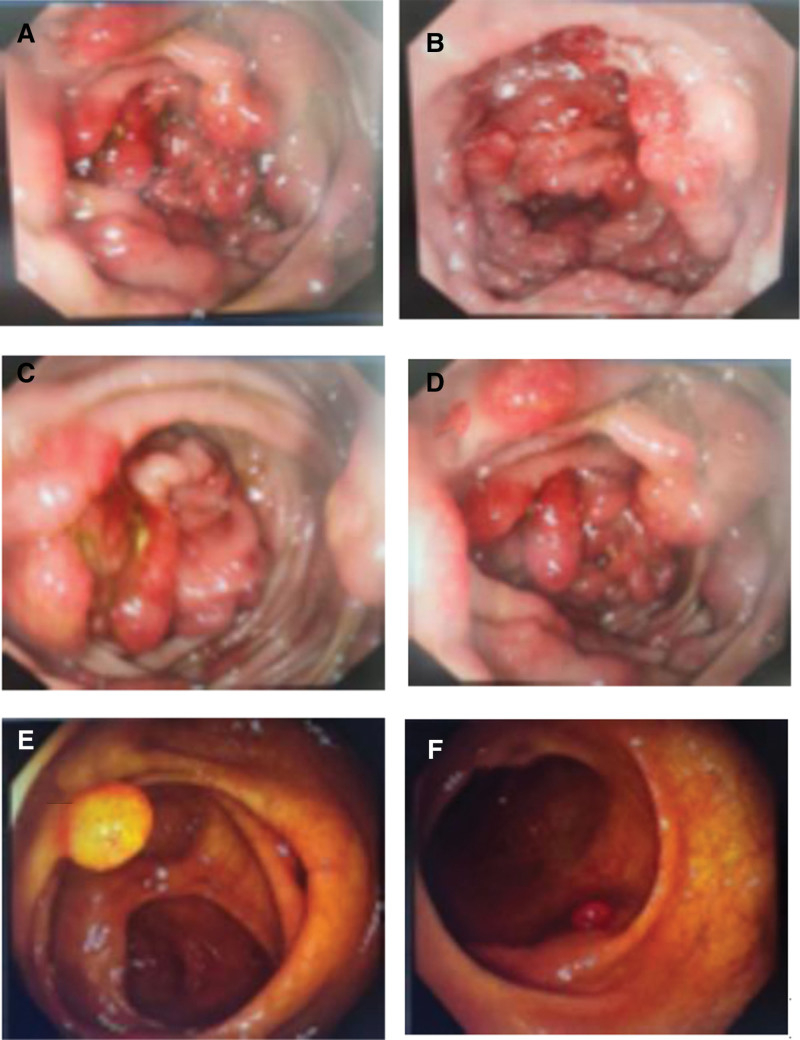
Colonoscopy before treatment revealed hundreds of congested, polypoid elevations throughout the colon and rectum, with edematous mucosa and loss of vascular pattern (A–D). Follow-up colonoscopy at 15 months showed a marked reduction in the number and size of colonic polyps (E and F).

**Figure 3.**
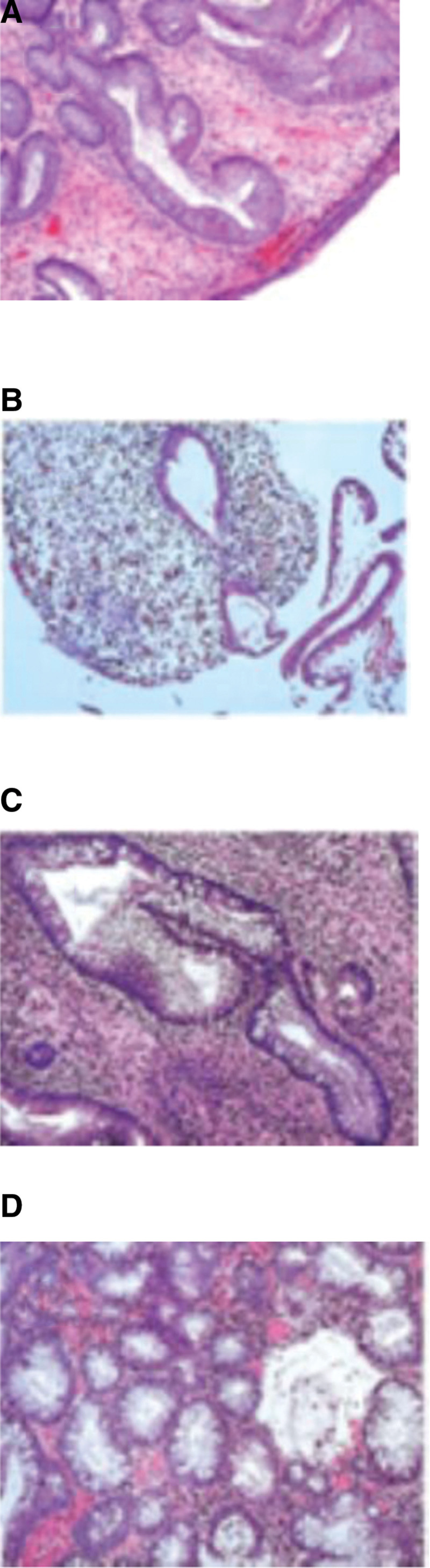
Pretreatment colonic biopsy showing dilated glands, interstitial inflammatory infiltrate, and vascular congestion (A). After 3 mo of treatment: glandular hyperplasia, cystic dilation, and marked congestion and edema in the lamina propria (B). At 8 mo: glandular hyperplasia with mild heterotopia, congestion, edema, and moderate chronic inflammation (C). At 15 mo: persistent glandular hyperplasia with mild to moderate cellular heterogeneity (D).

Gastroscopy was suggestive of congestion and edema of the mucosa of the fundus and body of the stomach, with red–white, extensive congestion and edema of the mucosa of the gastric horns and the gastric sinuses, uneven mucosal hyperplasia, and scattered patches of flattened erosions. Gastroscopic pathology showed mucosal glands without heterogeneity, adenoepithelial hyperplasia was seen focally, and interstitial chronic inflammatory cell infiltration. Colonoscopy showed hundreds of 0.3 to 2.0 cm polypoid elevations in the whole colon and rectum, with congested surface, congested and edematous mucosa of the cecum, and blurred vascular texture. (Fig. 2A–D). Pathology showed hyperplastic polyps in the descending colon as well as the rectum (Fig. 3A). Abdominal CT showed no definite thickening of the gastrointestinal tract wall.

### 2.5. Diagnosis and treatment

**Figure 4.**
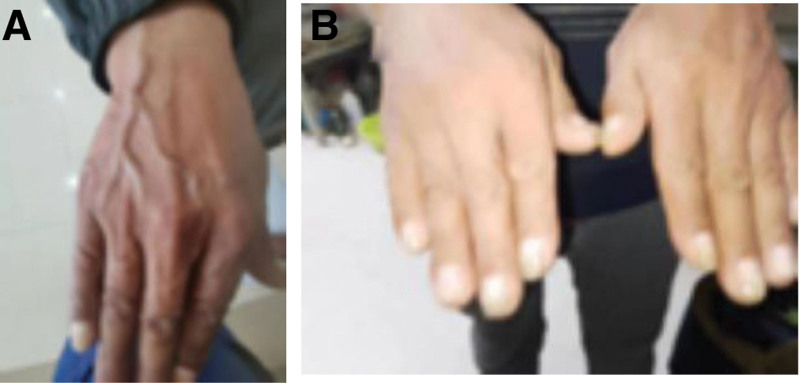
Restoration of normal skin pigmentation after treatment (A and B).

The diagnosis of CCS was made based on ectodermal abnormalities, diffuse polyps in the gastrointestinal tract, and pathology showing hyperplastic polyps. The patient received corticosteroid therapy (oral methylprednisolone tablets, 40 mg/day), salicylic acid analogs (mesalazine enteric-coated tablets, 3 grams/day), proton-pump inhibitors (rabeprazole sodium enteric-coated capsules, 20 mg/day), gastric mucoprotectants (Rebapatide tablets, 30 mg/day), calcium supplements (calcium carbonate D3 600 mg/day), and probiotics (Lactobacillus bifidus triplex 6 g/day). In the first week, the patient’s skin pigmentation improved significantly (Fig. 4A); in the second 3 weeks, the skin color returned to normal (Fig. 4B), hair growth resumed, and all laboratory parameters returned to normal. The patient was treated with a combination of oral corticosteroids and mesalazine for 14 months, with a corticosteroid dose of 40 mg/day for the first 3 months and a subsequent taper to 10 mg/day until discontinuation. The patient showed significant improvement in clinical symptoms, including improved appetite and weight gain.

### 2.6. Follow-up

The patient underwent regular follow-up for about 3 years and has been complying with the medication and endoscopic follow-up recommendations of the treating physician, with 4 endoscopies performed at intervals of about 3 months, 6 months, and 6 months in that order. This helped us to be able to more accurately assess the therapeutic effect of corticosteroids combined with mesalazine. Although the evolution of polyps in this patient followed the mucosal hyperplasia-adenoma pathway (Fig. 3B–D), the colonoscopic findings showed that both the size and the number of polyps were progressing in a favorable direction (Fig. 2E and F). 1 year later, the patient’s symptoms disappeared due to the active medication and endoscopy, and the medication was ended. After that, the patient’s condition was maintained well with no signs of recurrence at yearly follow-ups, and therefore, endoscopy was declined.

## 3. Discussion

CCS was first identified by Cronkhite and Canada in 1955. It is extremely rare, with only about 500 cases, of which about 70% are from Japan. The 5-year mortality rate is as high as 55%, and the majority of deaths have been associated with malnutrition, gastrointestinal hemorrhage, recurrent infections, sepsis, intestinal obstruction, and electrolyte disorders.^[3,4]^ However, recent studies have demonstrated a 5-year survival rate of 87.4%.^[5]^ Although its etiology and pathogenesis are unknown, it is generally accepted that CCS involves immune-related mechanisms, evidence of which includes Antinuclear Antibody (ANA) positivity, the finding of IgG4 plasma cell infiltration in some CCS polyps, and a favorable response to immunosuppressive agents.^[6]^ CCS has also been associated with a variety of autoimmune diseases: systemic lupus erythematosus, vitiligo, rheumatoid arthritis, scleroderma, and hypothyroidism.^[7,8]^ Several studies have found H. pylori infection to be associated with CCS.^[9]^ CCS resolves with anti-H. pylori therapy. Among other things, stress, fatigue, and vitamin deficiencies are among the causative factors of this disease.

CCS polyps have a tendency to become malignant, and the incidence of gastric or colon cancer can be 10% to 20%.^[10]^ And for avoiding fatal complications as well as the possibility of cancer, early detection and early treatment are necessary; therefore, detailed diagnostic criteria are necessary. CCS is typically characterized by the presence of diffuse polyps throughout the gastrointestinal (GI) tract, and the main pathological types of CCS polyps include inflammatory polyps, hyperplastic polyps, juvenile polyps, and adenomatous polyps.^[11]^ CCS usually manifests itself as diffuse upper GI tract inflammation and edema, thus predisposing endoscopy to misdiagnosis of other conditions rather than polyposis syndrome, as was the case in this case, where there were no polypoid lesions in the upper GI tract.^[12]^ When multiple polyps are present in the gastrointestinal tract, CCS usually needs to be differentiated from Peutz-Jeghers syndrome, juvenile polyposis syndrome, familial adenomatous polyposis, Turcot syndrome, Cowden syndrome, and other diseases.^[13]^ Among these, familial adenomatous polyposis is an inherited disease characterized by the presence of numerous adenomatous polyps in the colon and rectum, mostly without systemic manifestations, which significantly increases the risk of colorectal cancer.^[14]^ We performed endoscopy on the immediate family of this patient, and no abnormalities were seen in the colonic mucosa, so this disease was excluded. There have been many cases of malignant polyps following the adenoma-carcinoma sequence, and early detection and treatment are necessary; therefore, we recommend regular endoscopic follow-up in this patient.

Although there is no recognized standard in the treatment of this disease, long-term treatment with corticosteroids is effective, and for active CCS, high-dose hormone therapy, such as 30 to 49 mg/day for 6 to 12 months, is currently recommended. Statistically, the average recovery time for ectodermal changes is about 97 days, and the average time for regression of colonic polyps is 238 days.^[10]^ However, not all patients are suitable for this approach; recurrence may occur at later stages of steroid tapering, serious adverse effects can occur, and prolonged high-dose steroid therapy increases the risk of osteoporosis, diabetes mellitus, cardiovascular disease, and infections. Therefore, long-term treatment with small doses of steroids is safer.^[15]^ For steroid-dependent or steroid-resistant patients, azathioprine, fecal microbiota transplantation, and TNF-α antibody would be better alternatives.^[16–18]^ Significant efficacy has also been reported with lorazepam and mesalazine.^[19,20]^ The dose we chose has a high safety profile with reference to the therapeutic dose for ulcerative colitis.^[20,21]^ In this case, treatment with corticosteroids in combination with mesalazine resulted in a shorter recovery time from ectodermal abnormalities compared to corticosteroid-based treatment alone. In the initial treatment, the combination of proton-pump inhibitors and gastric mucosal protectors was aimed at alleviating the discomfort of the patient’s symptoms, and we believe that the efficacy of the combination of these 2 drugs was significant in the patient’s symptoms of gastroparesis.

Endoscopic evaluation has a great role in initial diagnosis and cancer surveillance, but endoscopy is not well suited for the evaluation of CCS remission or recurrence. Annual surveillance colonoscopy is recommended to assess mucosal disease activity and to remove adenomas and other precancerous lesions. The strength of this case report is the combination of long-term standardized symptom assessment with endoscopic follow-up.

## 4. Conclusion

In conclusion, due to the rarity of CCS, there is no recognized optimal treatment for this syndrome. However, corticosteroids combined with mesalazine drug therapy can relieve clinical symptoms and endoscopic manifestations in patients with CCS, while endoscopic mucosal resection surgery can be used as an adjunctive treatment. CCS has a relatively poor prognosis and carries a risk of cancer, so regardless of the use or discontinuation of steroid therapy, it is recommended that endoscopy should be performed every 3 to 6 months for 1 year after the first definitive diagnosis. After that, endoscopy is recommended every other year and continued for 3 years for effective cancer prevention.

## Author contributions

**Conceptualization:** Ruirui Yang.

**Investigation:** Ruirui Yang.

**Supervision:** Yufeng Li.

**Writing – original draft:** Ruirui Yang.

**Writing – review & editing:** Ruirui Yang, Wei Jiang, Yufeng Li.
